# Carbapenem-resistant Gram-negative pathogens: molecular epidemiology, diagnostic advances, and emerging therapeutic strategies

**DOI:** 10.3389/fmicb.2026.1913726

**Published:** 2026-08-18

**Authors:** Musharraf Khan, Praful Patil, Jaishriram Rathored

**Affiliations:** 1 Department of Microbiology, Jawaharlal Nehru Medical College, Datta Meghe Institute of Higher Education and Research (DMIHER), Wardha, India; 2 Central Research Laboratory and Molecular Diagnostics, School of Allied Health Sciences, Datta Meghe Institute of Higher Education and Research (DMIHER), Wardha, India

**Keywords:** antimicrobial stewardship, carbapenem resistance, carbapenemases, genomic surveillance, Gram-negative pathogens, molecular diagnostics, novel therapeutics

## Abstract

Carbapenem-resistant Gram-negative pathogens (CR-GNPs) have become an important global health problem, contributing significantly to healthcare-associated infections, extended hospital stays, high mortality rates, and higher healthcare costs. The dissemination of carbapenem resistance is mainly attributed to the spread of carbapenemase-encoding genes, such as the *Klebsiella pneumoniae* carbapenemase (KPC), the New Delhi metallo-β-lactamase (NDM), the Verona integron-encoded metallo-β-lactamase (VIM), the imipenemase (IMP), and the oxacillinase-48 (OXA-48)-like enzymes associated with clinically important Gram-negative pathogens, including *Klebsiella pneumoniae*, *Escherichia coli*, *Acinetobacter baumannii*, and *Pseudomonas aeruginosa*. As well as carbapenemase production, resistance can also develop via alteration of porins, upregulation of efflux pumps, and the buildup of several resistance factors, generating highly adaptable and hard-to-treat microbes. Phenotypic resistance patterns may not predict the underlying mechanism and accurate laboratory detection remains challenging. The identification and monitoring of carbapenem-resistant organisms have undergone improvement in recent years thanks to molecular diagnostics, rapid phenotypic tests, whole-genome sequencing and metagenomics. At the same time, new drugs have been developed, such as ceftazidime-avibactam, meropenem-vaborbactam, imipenem-relebactam, cefiderocol and combinations of aztreonam, offering increased treatment options, but with emerging resistance an issue. This mini review covers the molecular epidemiology of CR-GNPs, the latest developments and challenges in diagnosing these infections, new therapeutic options, and future perspectives on genomic surveillance, antimicrobial stewardship, and precision medicine strategies to address the increasing threat of carbapenem resistance.

## 1 Introduction

### 1.1 Background and clinical importance

Antimicrobial resistance (AMR) is one of the biggest threats to global public health and Gram-negative bacteria make a significant contribution to healthcare-associated infections and deaths due to AMR. Of these pathogens, CR-GNPs are of particular clinical importance as carbapenems are generally used as last-line therapy for treating severe infections with multidrug-resistant (MDR) organisms. Carbapenems are considered last-line agents for severe multidrug-resistant infections; however, their clinical utility is increasingly compromised by the global spread of carbapenem resistance, and resistance to these agents has been linked with worse outcomes such as longer hospital stays, higher health care costs, higher mortality (40% or more in severe invasive infections), and failure of treatment (Murray et al., 2022). Carbapenem-resistant Enterobacterales (CRE), carbapenem-resistant *Acinetobacter baumannii* (CRAB), and carbapenem-resistant *Pseudomonas aeruginosa* (CRPA) are considered critical-priority bacterial pathogens that need immediate research and development of new diagnostic and therapeutic options. They are being more frequently identified in bloodstream infections, ventilator-associated pneumonia (VAP), urinary tract infections, surgical site infections, and outbreaks in the intensive care unit (ICU) (Sati et al., 2025).

### 1.2 Resistance mechanisms

Their success is largely attributed to remarkable genomic plasticity, efficient acquisition of mobile genetic elements, and adaptation to healthcare environments. Carbapenem resistance is primarily mediated by the production of carbapenemases, enzymes capable of hydrolysing carbapenems and other β-lactam antibiotics. The main families of carbapenemases are *Klebsiella pneumoniae* carbapenemase (KPC), New Delhi metallo-β-lactamase (NDM), Verona integron-encoded metallo-β-lactamase (VIM), imipenemase (IMP), and oxacillinase-48 (OXA-48)-like enzymes (Tacconelli et al., 2018). These resistance determinants are often carried by mobile genetic elements such as plasmids, transposons and integrons, which allow for rapid spread in a range of bacterial species. In addition to carbapenemase production, resistance may result from porin loss, efflux pump overexpression, target modification, or the coexistence of multiple resistance mechanisms within a single isolate.

### 1.3 Global epidemiology

The epidemiology of carbapenem resistance has changed significantly in the last 10 years (Bonomo et al., 2018). In North America and some parts of Europe, the presence of KPC-producing Enterobacterales is prevalent, while NDM-producing Enterobacterales are widely distributed around South Asia, the Middle East and recently in other parts of the world. The spread of OXA-48-like carbapenemases has occurred worldwide throughout Europe, North Africa, and the Indian subcontinent. At the same time, internationally spread clones like *Klebsiella pneumoniae* ST258, ST11, ST147 and *Acinetobacter baumannii* Global Clone 2 have facilitated the global spread of resistance genes and made a significant contribution to many healthcare-associated outbreaks (HAIs). Rapid identification of carbapenem-resistant pathogens is still a great challenge. Traditional phenotypic susceptibility testing may have delayed results and may not be able to capture the underlying resistance mechanisms (Logan and Weinstein, 2017).

### 1.4 Diagnostics and treatment

Recent advances in rapid phenotypic assays, multiplex polymerase chain reaction (PCR), whole-genome sequencing (WGS), and metagenomic technologies have improved diagnostic accuracy and enhanced surveillance capabilities. However, these technologies remain underutilized in many low- and middle-income countries owing to limited laboratory infrastructure, financial constraints, and restricted access to advanced molecular diagnostics (Bonomo et al., 2018). Treatment of CR-GNP infections remains challenging. Novel β-lactam/β-lactamase inhibitor (BL/BLI) combinations like ceftazidime-avibactam, meropenem-vaborbactam and imipenem-relebactam are effective against selected carbapenemase-producing Enterobacterales (CPEs), but resistance to these combinations has already been reported. To overcome these limitations, new treatment options, such as cefiderocol, aztreonam combinations, bacteriophage therapy, and precision antimicrobial stewardship, are being evaluated (Sati et al., 2025).

### 1.5 Aim of the review

This Mini Review summarizes the current understanding of the molecular epidemiology of carbapenem-resistant Gram-negative pathogens, recent advances in molecular diagnostics and therapeutic strategies, and future directions, including genomic surveillance, precision diagnostics, and antimicrobial stewardship, to address the growing global challenge of carbapenem resistance.

## 2 Molecular epidemiology of CR-GNPs

The global emergence and dissemination of carbapenem-resistant Gram-negative pathogens (CR-GNPs) represent one of the greatest challenges in antimicrobial resistance (AMR). Carbapenem resistance is now a major public health threat in both healthcare and community settings. The spread of carbapenem-resistant organisms is influenced by mobile genetic elements, epidemic clones, international travel, healthcare-associated transmission, and antimicrobial selective pressure. Among Gram-negative bacteria, CRE, CRAB, and CRPA are the most clinically significant because they are associated with severe infections, limited treatment options, and high mortality rates (Wyres and Holt, 2018).

### 2.1 Major CR-GNPs

#### 2.1.1 Carbapenem-resistant Enterobacterales (CRE)

Globally, CRE have become major causes of bloodstream infections, urinary tract infections, ventilator-associated pneumonia (VAP), and intra-abdominal infections. *Klebsiella pneumoniae* is the most common species of CRE and is a significant reservoir of carbapenemase genes. Clonal lineages of *K. pneumoniae* have been very successful in spreading globally, especially ST258, ST11, ST147, ST15, and ST307, which show significant ability to acquire AMR and to spread nosocomially. Carbapenem resistance determinants have also increasingly emerged in *E. coli*, *Enterobacter cloacae* complex, *Citrobacter freundii*, *Serratia marcescens* and *Klebsiella oxytoca* (Van Duin et al., 2020). Plasmids containing the genes for *Klebsiella pneumoniae* carbapenemase (KPC), New Delhi metallo-β-lactamase (NDM), Verona integron-encoded metallo-β-lactamase (VIM), imipenemase (IMP) and OXA-48-like enzymes play a major role in the dissemination of carbapenemase-producing Enterobacterales (CPEs). These resistance determinants are often in tandem with resistance to aminoglycosides, fluoroquinolones and polymyxins, leading to extensively drug-resistant phenotypes (David et al., 2019).

#### 2.1.2 Carbapenem-resistant *Acinetobacter baumannii* (CRAB)

*Acinetobacter baumannii* is now a major cause of healthcare-associated infection, especially in the ICU. Its ability to survive on environmental surfaces and acquire resistance determinants has contributed substantially to its global dissemination. The OXA-type of carbapenemases, especially OXA-23, OXA-24/40, OXA-58, and OXA-143, are the predominant types of carbapenemases associated with *A. baumannii*. International Clone 2 (IC2), or Global Clone 2, is the most common epidemic lineage causing many of the current outbreaks around the world. Carbapenem-resistant *A. baumannii* is still considered a critical-priority pathogen by the World Health Organization, as therapeutic options remain limited and the mortality rate is often higher than 50% among patients admitted to intensive care units (Lam et al., 2023).

#### 2.1.3 Carbapenem-resistant *Pseudomonas aeruginosa* (CRPA)

*Pseudomonas aeruginosa* is intrinsically resistant to several antimicrobial classes and readily acquires additional resistance during therapy. *P. aeruginosa* carbapenem resistance is often facilitated by several mechanisms, including OprD porin loss, production of metallo-β-lactamases (MBLs) and upregulation of multidrug efflux pumps. Internationally disseminated high-risk clones of *P. aeruginosa*, including ST111, ST175, ST235, and ST244, have contributed substantially to the global spread of carbapenem resistance. *P. aeruginosa* is an extremely versatile organism with multiple resistance mechanisms that can occur in a single isolate, making both traditional and new antimicrobials ineffective against it (Oliver et al., 2015).

### 2.2 Global dissemination of carbapenemase genes

Carbapenem resistance has a global distribution where certain families of carbapenemases dominate in a given area. KPC enzymes are the most common carbapenemases found across North America, South America, Greece, Italy and in some European nations. KPC-producing *K. pneumoniae* has been endemic in many health-care systems and has caused numerous outbreaks worldwide since its first identification in the United States. In contrast, NDM enzymes have become widespread throughout the South Asian region, especially in India, Pakistan, Bangladesh and its neighboring countries. Highly transferable plasmids and international travel have contributed to the widespread dissemination of NDM-producing Enterobacterales. NDM genes have been reported on every continent. OXA-48-like carbapenemases are now widespread across Europe, Africa, the Middle East and the Indian subcontinent. However, the OXA-48 producers may exhibit relatively low carbapenem MICs, making laboratory detection difficult and facilitating silent dissemination. VIM and IMP carbapenemases are still present worldwide, but are not as common as KPC, NDM and OXA-48. They continue to be significant contributors to healthcare-associated outbreaks, however, especially with *P. aeruginosa* and Enterobacterales isolates (Al Souheil et al., 2025).

### 2.3 Mobile genetic elements and horizontal gene transfer

Plasmids, transposons, insertion sequences, and integrons facilitate horizontal gene transfer, enabling rapid dissemination of carbapenem resistance determinants within and across bacterial species. Carbapenemase genes are often carried on conjugative plasmids with other resistance factors, giving rise to multi-drug and extensively drug-resistant variants (Partridge et al., 2018). Recent genomic studies have demonstrated the development of hybrid plasmids that encode AMR and virulence. These convergent plasmids facilitate the emergence of highly pathogenic and difficult-to-treat *K. pneumoniae* lineages. The rising number of these hybrid elements points to the necessity of coupled genomic surveillance programs to track resistance as well as virulence evolution (Chen et al., 2024). The continued emergence of highly transmissible plasmids and epidemic clones underscores the need for coordinated genomic surveillance, infection prevention, and antimicrobial stewardship (Wyres et al., 2020).

## 3 Molecular mechanisms of carbapenem resistance

The emergence and rapid spread of CR-GNPs are largely due to the variety of molecular mechanisms that reduce or abolish the activity of carbapenem antibiotics. Carbapenemase production is the most clinically relevant mechanism; however, resistance often results from a combination of enzymatic and non-enzymatic mechanisms such as outer membrane porin changes, multidrug efflux pump overexpression, target modification and accumulation of multiple resistance determinants in a single isolate. These mechanisms are not mutually exclusive, and their presence adds to the complexity of the resistance level and of the diagnosis, surveillance and therapeutic management (Nordmann and Poirel, 2019; Yuan et al., 2024).

### 3.1 Carbapenemase production: the dominant resistance mechanism

Carbapenemase production is the predominant mechanism of carbapenem resistance among CR-GNPs. Carbapenemases are β-lactamases that hydrolyse carbapenems and most other β-lactam antibiotics. These enzymes are grouped by the Ambler molecular classification system as types A, B and D. These genes are frequently located on transferable plasmids and mobile genetic elements, enabling the fast horizontal transfer between bacteria (Li C. et al., 2024; Queenan and Bush, 2007).

#### 3.1.1 Class A carbapenemases: *Klebsiella pneumoniae* carbapenemase (KPC)

*Klebsiella pneumoniae* carbapenemase enzymes are the most widespread class A carbapenemases and have caused numerous hospital outbreaks worldwide. Since the first description of KPC in the United States, KPC-producing Enterobacterales have become endemic in many countries. The blaKPC gene is usually found on transposon Tn4401 and is part of highly transmissible plasmids that enable interspecies transfer. The KPCs are highly active against penicillins, cephalosporins, monobactams and carbapenems. High-risk clones, like *Klebsiella pneumoniae* ST258 and ST11, have contributed to their global spread. Notably, newer β-lactamase inhibitors–namely, avibactam, vaborbactam and relebactam–can inhibit KPC enzymes as well, making them critical therapeutic targets (Van Duin and Doi, 2017).

#### 3.1.2 Class B carbapenemases: MBLs

Metallo-β-lactamases represent one of the greatest therapeutic challenges in modern clinical microbiology. There are three major types of MBL family: New Delhi metallo-β-lactamase (NDM), Verona integron-encoded metallo-β-lactamase (VIM), and imipenemase (IMP). Since its first description in 2008, NDM has disseminated globally. The distribution of NDM-producing organisms is especially widespread across South Asia, the Middle East and Africa, but has been reported globally. Particularly, the MBLs are not inhibited by the currently available β-lactamase inhibitors, including avibactam, vaborbactam and relebactam. Multiple resistance determinants have been carried on highly mobile IncF, IncA/C and IncX plasmids, facilitating the spread of NDM enzymes. Hence, aminoglycosides (such as amikacin or gentamicin), fluoroquinolones, and polymyxins remain generally ineffective, severely restricting therapy against NDM-producing isolates (Boyd et al., 2020).

#### 3.1.3 Class D carbapenemases: OXA-type enzymes

The OXA-type carbapenemases are a large class of oxacillin-hydrolysing β-lactamases that have emerged as a significant group of mobile enzymes in worldwide use. OXA-48-like enzymes are common among Enterobacterales, while OXA-23, OXA-24/40 and OXA-58 are more common in *Acinetobacter baumannii*. A major challenge associated with OXA-48-producing organisms is the variability of their resistance phenotype. Some isolates show only small rises in carbapenem MICs and hence are not detected by routine testing. Consequently, these isolates may spread silently within healthcare settings, complicating infection control (Poirel et al., 2012).

### 3.2 Porin loss and reduced membrane permeability

Reduced outer membrane permeability also contributes significantly to carbapenem resistance alongside carbapenemase production. Porin proteins are needed for antibiotics to enter the Gram-negative bacterial periplasmic space. Mutations, deletions or downregulation of porin genes decrease antibiotic entry and antimicrobial activity. For *K. pneumoniae*, the loss of the porins OmpK35 and OmpK36 has been closely linked to carbapenem resistance. Likewise, *Pseudomonas aeruginosa* commonly acquires resistance by inactivating its porin protein OprD, which is the main imipenem-importing protein. Some cases of low-level resistance may be caused by porin deficiency alone, but when it is coupled with extended-spectrum β-lactamases (ESBLs) or AmpC β-lactamases (AmpC), clinically important carbapenem resistance can develop. Many isolates therefore have both enzyme-mediated and permeability-mediated resistance mechanisms (Maher and Hassan, 2023).

### 3.3 Multidrug efflux pumps

Efflux pumps belong to the active efflux mechanisms that actively extrude antimicrobial agents from bacterial cells and play a significant role in the development of multidrug resistance among several Gram-negative pathogens. Such transport systems diminish intracellular antibiotic levels and frequently function in concert with other resistance mechanisms. The MexAB-OprM, MexXY, and MexCD-OprJ systems are found in *P. aeruginosa* and are crucial to carbapenem resistance. *Acinetobacter baumannii* contains several efflux pumps of the resistance-nodulation-division (RND) family, such as AdeABC, AdeFGH, and AdeIJK, that are responsible for its broad-spectrum AMR. Efflux pump overexpression alone rarely results in high-level carbapenem resistance, but in combination with carbapenemase production or porin loss can lead to high-level resistance (Li et al., 2015).

### 3.4 Coexistence of multiple resistance determinants

A defining feature of contemporary carbapenem resistant pathogens is the presence of multiple resistance mechanisms in single isolates. The clinical strains currently identified more frequently carry ESBLs, AmpC, porin mutations, efflux pump overexpression and resistance determinants to aminoglycosides, fluoroquinolones, tetracyclines and polymyxins, in addition to the carbapenemases. The presence of blaNDM in combination with mcr genes, which encode resistance to colistin, a last-resort antibiotic, is a particular concern as it restricts the use of both first-line and last-resort antibiotics. Indeed, the blaKPC, blaOXA-48, and blaNDM genes are often accompanied by other plasmid-mediated resistance genes, conferring extensively drug-resistant or pan-drug-resistant phenotypes. These multidimensional resistance patterns severely limit therapeutic options and increase the risk of treatment failure (Grossman and Thompson, 2026).

### 3.5 Emergence of resistance–virulence convergence

Recent genomic studies have identified hybrid plasmids carrying both antimicrobial resistance and virulence determinants. This phenomenon has been most extensively described in *Klebsiella pneumoniae* (KP), where hypervirulent strains have gained plasmids containing carbapenemase genes and resistant clones have gained virulence plasmids. The convergence of resistance and virulence promotes the emergence of highly virulent, multidrug-resistant strains capable of causing invasive infections. These convergent pathotypes are a growing danger due to their combination of greater transmissibility, pathogenicity, and resistance to therapy. Together, the mechanisms of carbapenem resistance highlight the remarkable adaptability of Gram-negative pathogens. The continued spread of resistance is driven by the interplay between carbapenemase production, altered membrane permeability, efflux systems, and mobile genetic elements. Understanding these mechanisms is essential for developing accurate diagnostics, effective therapies, and robust surveillance programs (Li et al., 2025).

**FIGURE 1:**
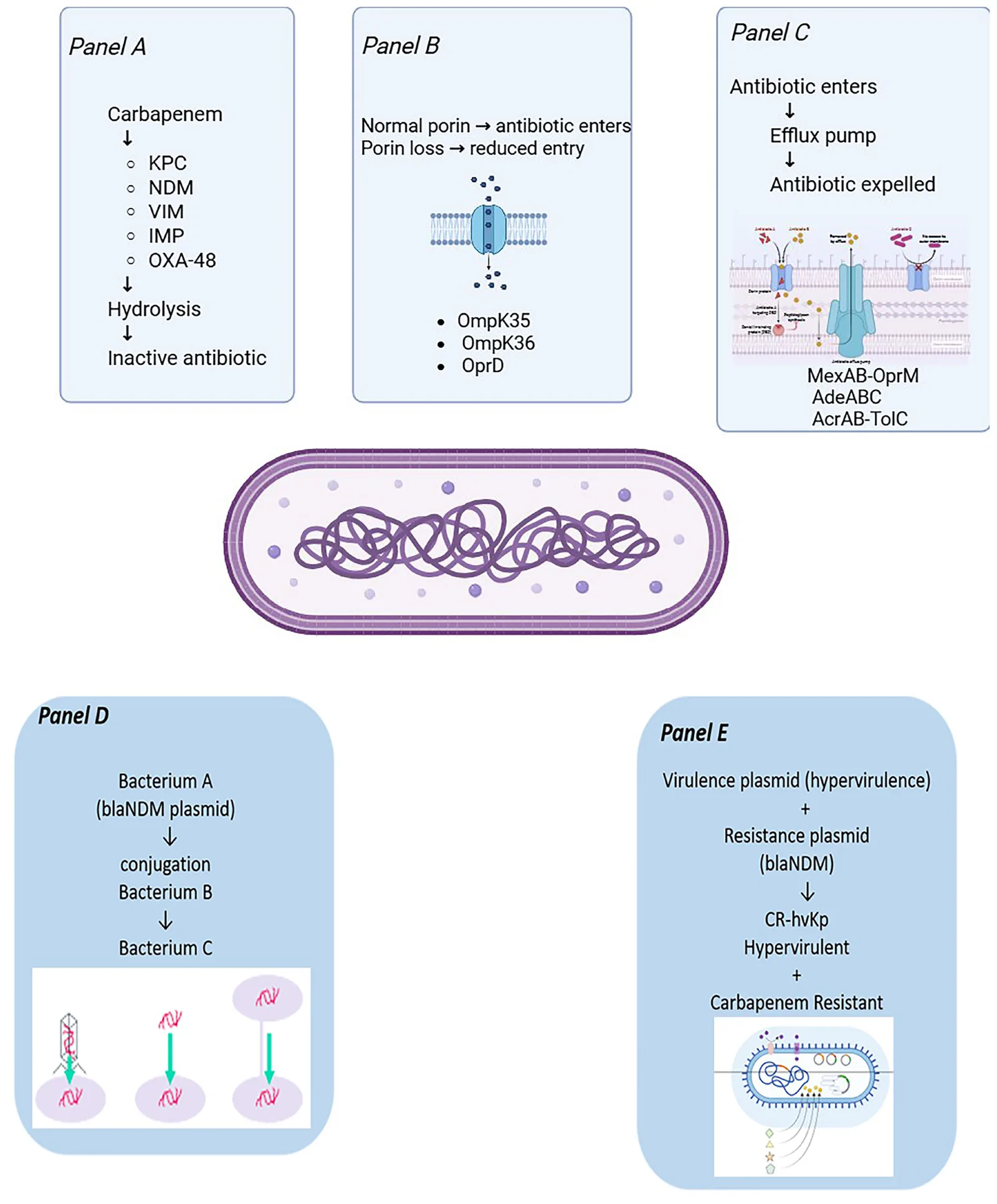
Major molecular mechanisms and dissemination pathways of carbapenem resistance in Gram-negative pathogens. Carbapenem resistance is mediated by panel **(A)** carbapenemase production, **(B)** porin loss or modification, **(C)** efflux pump overexpression, and **(D)** horizontal gene transfer through mobile genetic elements. **(E)** Global dissemination is facilitated by healthcare transmission, international travel, environmental reservoirs, and plasmid-mediated spread of resistance genes.

The major mechanisms contributing to carbapenem resistance and their dissemination pathways are summarized in Figure 1.

## 4 Diagnostic advances and current challenges

The increasing prevalence of CR-GNPs highlights the need for rapid, accurate, and cost-effective diagnostic methods. Rapid detection of any carbapenem resistance is critical to promptly begin appropriate antimicrobial treatment, infection prevention, and epidemiological surveillance. Laboratory diagnosis remains challenging because carbapenem resistance arises through multiple mechanisms, including carbapenemase production, porin alterations, efflux pump overexpression, or combinations of these mechanisms. Moreover, resistance phenotypes are not necessarily associated with the molecular mechanism, underscoring the need for a combination of phenotypic and molecular diagnostic strategies (Schuetz et al., 2025).

### 4.1 Conventional phenotypic susceptibility testing

Routine antimicrobial susceptibility testing (AST) remains the backbone of the detection of carbapenem resistance in clinical microbiology laboratories. Carbapenem susceptibility is routinely determined using disk diffusion, broth microdilution, automated susceptibility systems, and gradient diffusion methods. Although these methods are widely used and inexpensive, they have important limitations. Minimum inhibitory concentrations (MICs) of carbapenems may vary according to the underlying resistance mechanism; some carbapenemase-producing isolates, especially those that produce OXA-48, may have borderline susceptibility results. Thus, using standard, non-molecular susceptibility testing alone can result in under-recognition of clinically important CPEs. In addition, phenotypic AST typically takes 18–24 h after the bacteria have been isolated, which means that therapeutic decisions and infection control measures are delayed. These limitations have driven the development of rapid carbapenemase detection assays for CPEs (Simner et al., 2024).

### 4.2 Rapid phenotypic carbapenemase detection assays

Several phenotypic tests have been developed for rapid detection of carbapenemase production.

#### 4.2.1 Modified Carbapenem Inactivation Method (mCIM)

The Clinical and Laboratory Standards Institute (CLSI) now recommends the mCIM as a reliable phenotypic screening test for carbapenemase production. The assay measures the capacity of the test organism to inactivate carbapenems and is very sensitive and specific for most clinically relevant carbapenemases. Its benefits are its simplicity, low cost and ease of use in resource-poor laboratories. However, the assay cannot identify the specific class of carbapenemase responsible for resistance (Pierce et al., 2017).

#### 4.2.2 Carba NP test

The Carba NP test detects antibiotic hydrolysis by measuring pH changes resulting from carbapenem degradation. This test can yield results in less than 2 h and is highly sensitive for KPC, NDM, VIM and IMP enzymes. However, OXA-type carbapenemases and some *Acinetobacter* species have been shown to have some variability in performance despite the short turnaround time. Further, the preparation of reagents and interpretation may be technically challenging in the routine diagnostic environment (Simner et al., 2024).

#### 4.2.3 Lateral flow immunochromatographic assays

In recent years, lateral flow assays have emerged to facilitate the direct detection of most carbapenemases, such as KPC, NDM, VIM, IMP and OXA-48. Such assays provide results within 15–30 min and are highly accurate for diagnosis. Lateral flow assays are widely used in clinical microbiology because they are simple, require minimal technical expertise, and enable rapid confirmation of carbapenemase-producing organisms (Bernabeu et al., 2022).

### 4.3 Molecular diagnostics

Molecular approaches to the detection of CR-GNPs have revolutionized the way we diagnose resistance, allowing direct detection of resistance genes in the causative organism.

#### 4.3.1 Conventional and multiplex PCR

Polymerase chain reaction remains one of the most widely used molecular methods for the identification of carbapenemase. Specific primers that detect blaKPC, blaNDM, blaVIM, blaIMP, and blaOXA-48 genes can be used to quickly and sensitively identify major determinants of resistance. Multiplex PCR platforms have additionally made it easier to identify multiple carbapenemase genes in one test. These types of assays are especially useful in areas with more than one family of carbapenemase present. PCR-based methods, however, are only useful if resistance genes are known and do not allow the detection of novel or previously uncharacterized resistance mechanism (Mayta-Barrios et al., 2025).

#### 4.3.2 Real-time PCR

Carbapenemase genes can be detected and quantified with excellent analytical sensitivity and quickly using real-time PCR. Some commercial assays permit direct testing from clinical specimens, reducing the time to appropriate therapeutic intervention. Many low- and middle-income countries (LMICs) have a high prevalence of carbapenem resistance, but the cost of the instrumentation and reagents is also an obstacle to widespread adoption in these regions (Lutgring and Limbago, 2016).

#### 4.3.3 Loop-mediated isothermal amplification (LAMP)

Loop-mediated isothermal amplification has become an appealing alternative to PCR because, under isothermal conditions, no special thermal cyclers are necessary for amplification. Several studies have shown that the genes of NDM, KPC and OXA-48 can be detected by the LAMP-based assays. LAMP is uniquely well-suited for decentralized laboratories and resource-limited environments due to its simplicity, speed, and minimal equipment demands (Wang et al., 2021).

### 4.4 Whole-genome sequencing and genomic surveillance

Whole-genome sequencing (WGS) has transformed the surveillance of carbapenem-resistant Gram-negative pathogens (CR-GNPs) by enabling comprehensive characterization of antimicrobial resistance determinants and transmission dynamics. WGS enables simultaneous identification of carbapenemase genes (e.g., blaKPC, blaNDM, blaOXA-48, blaVIM, and blaIMP), virulence determinants, plasmid structures, mobile genetic elements, and clonal relationships in CR-GNPs, unlike targeted molecular assays. WGS has greatly enhanced outbreak investigations by tracing the transmission of high-risk clones of *Klebsiella pneumoniae, Pseudomonas aeruginosa*, and *Acinetobacter baumannii*, thereby supporting infection prevention and genomic surveillance programs. Genomic studies have also contributed to the understanding of the global dissemination of carbapenemase-producing organisms and resistance-virulence convergence. Despite these advantages, routine implementation of WGS for CR-GNP surveillance remains limited by sequencing costs, the need for specialized bioinformatics expertise, infrastructure requirements, and challenges in genomic data interpretation. Continued reductions in sequencing costs and advances in bioinformatics are expected to facilitate the integration of WGS into routine surveillance programs for CR-GNPs, enabling earlier detection of emerging carbapenem-resistant clones and strengthening infection prevention strategies (Köser et al., 2014; Sherry et al., 2025).

### 4.5 Metagenomics and culture-independent diagnostics

Metagenomic sequencing is an emerging technology for CR-GNP diagnosis and comprehensive AMR profiling. Metagenomics can directly detect CR-GNPs and carbapenemase genes (e.g., blaKPC, blaNDM, blaOXA-48, blaVIM, blaIMP) in clinical samples, whereas traditional culture-based methods need more time for detection. This approach enables identification of polymicrobial infections, uncultivable organisms, and the complete resistome of a sample, making it useful in HAIs, respiratory tract infections and bloodstream infections caused by CR-GNPs. Moreover, metagenomic sequencing has demonstrated great promise in the context of hospital outbreaks for the swift identification of carbapenem-resistant pathogens and as a tool for antimicrobial stewardship for early detection of clinically relevant resistance determinants. The clinical use is limited, however, because of the high costs, the absence of standard analytical workflows, contamination control, host DNA interference, and difficulties in the interpretation of the complex sequencing data. Improvements in technology and bioinformatics pipelines will likely continue to make metagenomics a useful tool for future CR-GNP diagnostics and surveillance programs (Gu et al., 2019; Olsen and Riber, 2025).

### 4.6 Artificial intelligence (AI) and digital microbiology

Artificial intelligence is rapidly emerging as a key tool for the diagnosis and surveillance of CR-GNPs. Incorporating antimicrobial susceptibility testing (AST) results with genomic data and clinical information, machine learning (ML) algorithms can help predict the mechanism of carbapenem resistance and aid in timely therapeutic decision-making. AI-based tools have shown good results in detecting CR-OGs, predicting the presence of carbapenemase genes, and automating genomic analysis to improve outbreak detection and genomic surveillance of CR-GNPs. AI image recognition systems can also accurately analyze culture plates and AST results, shorten the turnaround time and improve efficiency in the laboratory. Despite these advances, data standardization, model validation, algorithm transparency, and integration to routine clinical workflows remain to be a challenge to widespread implementation. The potential of AI to enhance early identification of CR-GNPs, support antimicrobial stewardship, and bolster infection prevention and control measures holds promise for future advancements. Future integration of AI with rapid molecular diagnostics, WGS, and real-time surveillance systems is expected to improve CR-GNP detection, antimicrobial stewardship, and infection prevention (Li Y. et al., 2024; Olatunji et al., 2024; Peiffer-Smadja et al., 2020).

### 4.7 Current challenges and future directions

Despite significant advances in diagnostic technologies, several challenges remain in the detection and management of CR-GNPs (is depicted in Table 1). No single diagnostic platform can comprehensively identify all carbapenem resistance mechanisms while simultaneously providing rapid turnaround, high accuracy, affordability, and broad accessibility. Although phenotypic methods remain indispensable for routine antimicrobial susceptibility testing, they often fail to accurately characterize the underlying resistance mechanisms. Molecular assays enable rapid detection of known carbapenemase genes but are limited in identifying novel or emerging resistance determinants. Likewise, WGS and metagenomic sequencing provide comprehensive genomic characterization of CR-GNPs but remain constrained by high costs, infrastructure requirements, specialized bioinformatics expertise, and challenges in clinical data interpretation. Therefore, integrating conventional susceptibility testing, rapid carbapenemase detection assays, targeted molecular diagnostics, WGS, metagenomics, and AI-assisted analytical platforms may represent the most effective strategy for the diagnosis, surveillance, and management of CR-GNPs. Future efforts should focus on developing affordable point-of-care (POC) molecular diagnostics, expanding access to sequencing technologies, improving AI-driven resistance prediction models, and strengthening genomic surveillance networks to enable timely diagnosis, effective infection prevention, antimicrobial stewardship, and optimized therapeutic management of CR-GNP infections (Sherry et al., 2025).

**TABLE 1:** Diagnostic approaches for CR-GNPs.

Diagnostic method	Target	Turnaround time	Advantages	Limitations
Disk diffusion/MIC	Phenotypic resistance	18–24 h	Widely available	Cannot identify mechanism
mCIM	Carbapenemase activity	18–24 h	Inexpensive, CLSI recommended	No enzyme classification
Carba NP	Carbapenem hydrolysis	2 h	Rapid	Reduced sensitivity for some OXA enzymes
Multiplex PCR	Resistance genes	2–6 h	High sensitivity	Detects only known genes
Real-time PCR	Resistance genes	1–4 h	Rapid and quantitative	High cost
LAMP	Resistance genes	<1 h	Suitable for low-resource settings	Limited multiplexing
WGS	Entire genome	24–72 h	Comprehensive characterization	Expensive, bioinformatics needed
Metagenomics	Pathogens + resistome	24–48 h	Culture-independent	Cost and data complexity
AI-assisted platforms	Resistance prediction	Variable	Precision surveillance	Still emerging

## 5 Emerging therapeutic strategies

Management of CR-GNP infections remains a major clinical challenge because of limited therapeutic options and increasing antimicrobial resistance. Treatment selection should be guided by the underlying resistance mechanism, infection site, disease severity, pharmacokinetic/pharmacodynamic considerations, and local epidemiology (Bassetti et al., 2021).

### 5.1 Novel BL/BLI combinations

Novel BL/BLI combinations have expanded treatment options for carbapenem-resistant infections.

#### 5.1.1 Ceftazidime-avibactam

Ceftazidime–avibactam is active against KPC- and OXA-48-producing Enterobacterales and has demonstrated improved clinical outcomes with lower nephrotoxicity than colistin-based regimens. However, it lacks activity against MBL-producing isolates (Yu et al., 2024).

#### 5.1.2 Meropenem-vaborbactam

Meropenem–vaborbactam restores meropenem activity against KPC-producing Enterobacterales and has shown excellent efficacy in bloodstream, urinary tract, and hospital-acquired infections, but it is ineffective against MBL producers (Bassetti et al., 2019).

#### 5.1.3 Imipenem-relebactam

Imipenem–relebactam is effective against KPC-producing Enterobacterales and selected MDR *Pseudomonas aeruginosa* isolates but has limited activity against MBL-producing organisms (Motsch et al., 2020).

### 5.2 Therapeutic challenges posed by MBLs

Metallo-β-lactamase-producing organisms, particularly those harboring blaNDM, remain difficult to treat because currently available BL/BLI combinations do not inhibit these enzymes (Falcone et al., 2021).

#### 5.2.1 Aztreonam-based therapy

Aztreonam is chemically resistant to hydrolysis by MBLs. Many ESBL or AmpC (aztreonam-inactivating) producing isolates, however, also co-express ESBLs and/or AmpC enzymes that inactivate aztreonam. The combination of aztreonam and ceftazidime-avibactam is effective because avibactam inhibits co-produced AmpC and ESBL enzymes, while aztreonam remains active against MBLs. This combination is a preferred treatment for severe infections caused by NDM-producing Enterobacterales (Zhang et al., 2020).

#### 5.2.2 Emerging MBL inhibitors

Several investigational MBL inhibitors targeting NDM, VIM, and IMP enzymes are under development; however, clinical evidence remains limited (Jalde and Choi, 2020).

### 5.3 Cefiderocol: a siderophore cephalosporin

Cefiderocol is a siderophore cephalosporin with broad activity against KPC-, NDM-, VIM-, and IMP-producing Enterobacterales, MDR *Pseudomonas aeruginosa*, and *Acinetobacter baumannii*. Although resistance has emerged, cefiderocol remains an important therapeutic option for the treatment of extensively drug-resistant CR-GNP infections (Parsels et al., 2021).

### 5.4 Combination antimicrobial therapy

Common combinations include:

- Polymyxin-based combinations
- Carbapenem-containing regimens
- Tigecycline combinations
- Aminoglycoside combinations
- Aztreonam plus ceftazidime-avibactam

Recent evidence suggests that targeted therapy with newer antimicrobial agents may be superior to routine combination therapy in selected patients. Combination therapy, however, could still be useful in the severely ill patient, infections with very resistant organisms, and when susceptibility data is unavailable. Therapy should then be tailored to the individual based on the severity of the infection, resistance mechanisms and antimicrobial susceptibility profiles (Paul et al., 2014).

### 5.5 Management of carbapenem-resistant *Acinetobacter baumannii* (CRAB)

Management of CRAB remains challenging because of limited therapeutic options and increasing resistance.

The current treatment algorithms typically include:

- Sulbactam-containing regimens
- Sulbactam-durlobactam combinations
- Cefiderocol
- Polymyxins
- Tigecycline
- Minocycline

Recent clinical studies identify sulbactam–durlobactam as a promising treatment for CRAB infections. Durlobactam inhibits *Acinetobacter* β-lactamases, increasing the activity of sulbactam, and is more effective than standard polymyxin drugs (Iovleva et al., 2025).

### 5.6 Management of carbapenem-resistant *Pseudomonas aeruginosa* (CRPA)

Carbapenem-resistant *Pseudomonas aeruginosa* (CRPA) remains one of the most challenging Gram-negative pathogens because of its intrinsic resistance mechanisms, remarkable genomic adaptability, and ability to acquire additional resistance determinants, including carbapenemases, overexpression of AmpC β-lactamase, efflux pump activation, and porin loss. Consequently, treatment should be guided by antimicrobial susceptibility testing and local resistance epidemiology (Tamma et al., 2023). Recent β-lactam/β-lactamase inhibitor combinations have expanded therapeutic options. Ceftolozane–tazobactam demonstrates excellent activity against many multidrug-resistant CRPA isolates lacking carbapenemases, whereas ceftazidime–avibactam and imipenem–relebactam are effective against selected KPC-producing and other susceptible isolates. Cefiderocol has also emerged as an important therapeutic option because of its potent activity against many difficult-to-treat CRPA strains, including isolates resistant to multiple antimicrobial classes (Bassetti et al., 2021). Although polymyxins and aminoglycosides remain alternative agents, their use is generally reserved for situations in which preferred therapies are unavailable or inactive because of concerns regarding toxicity and variable clinical efficacy. Optimal management of CRPA requires rapid identification of resistance mechanisms, susceptibility-guided antimicrobial selection, effective source control, and antimicrobial stewardship to optimize clinical outcomes and limit further emergence of resistance (Vidal-Cortés et al., 2025).

### 5.7 Bacteriophage therapy and future antimicrobial approaches

The rapid evolution of AMR has renewed interest in alternative therapeutic strategies.

#### 5.7.1 Bacteriophage therapy

Bacteriophages are viruses that specifically infect and kill bacteria. Personalized phage cocktails are effective in several compassionate-use cases and early clinical trials of MDR *Acinetobacter baumannii*, *Klebsiella pneumoniae*, and *Pseudomonas aeruginosa* infections. Despite encouraging early clinical experience, regulatory challenges, phage selection, resistance development, and manufacturing standardization remain important barriers to widespread clinical implementation (Gordillo Altamirano and Barr, 2019).

#### 5.7.2 Antimicrobial peptides and immune-based therapies

Antimicrobial peptides, monoclonal antibodies, anti-virulence products and host-directed agents are being evaluated to create additional options for treating carbapenem-resistant pathogens. These methods can be used to minimize selective pressure for resistance and maximize pathogen clearance (Mookherjee et al., 2020).

#### 5.7.3 Precision antimicrobial stewardship

Precision antimicrobial stewardship is emerging with the development of genomics, rapid diagnostics, and AI. A combination of AMR detection and personalized antimicrobial treatment algorithms can maximize antimicrobial selection and reduce the emergence of AMR, with the potential to optimize clinical outcomes (Latif et al., 2026).

### 5.8 Therapeutic outlook

Recent advances in BL/BLI combinations, cefiderocol, and precision antimicrobial strategies have substantially improved the management of CR-GNP infections (see in Table 2). However, the effectiveness of therapy is still critically reliant on early diagnosis, resistance mechanism characterization, source control and antimicrobial stewardship. Resistance continues to evolve, and precision medicine strategies that combine the use of genomic data, rapid diagnostics, and targeted antimicrobial selection will be the key methods used in the future treatment of resistant infections. Together, these advances provide new opportunities to enhance the care and outcomes of patients with carbapenem-resistant infections, but ongoing surveillance and innovation are needed to meet the evolving and growing challenge of CR-GNPs (Yin et al., 2023).

**TABLE 2:** Emerging therapeutic agents against CR-GNPs.

Agent	KPC	NDM	OXA-48	CRPA	CRAB
Ceftazidime-avibactam	✓	✗	✓	Variable	✗
Meropenem-vaborbactam	✓	✗	✗	Limited	✗
Imipenem-relebactam	✓	✗	✗	✓	✗
Cefiderocol	✓	✓	✓	✓	✓
Aztreonam + ceftazidime–avibactam	✓	✓	Variable	Limited	✗
Sulbactam-durlobactam	✗	✗	✗	✗	✓

## 6 Future perspectives

Despite major advances in diagnostics and therapeutics, CR-GNPs remain a significant global health threat because of the continued emergence of resistance mechanisms and high-risk clones. Future control strategies will rely on genomic surveillance, precision diagnostics, AI-assisted clinical decision support, antimicrobial stewardship, and One Health interventions.

### 6.1 Expansion of genomic surveillance programs

Routine implementation of WGS is expected to transform CR-GNP surveillance by enabling comprehensive characterization of resistance determinants, virulence factors, mobile genetic elements, and transmission pathways. Real-time genomic surveillance will facilitate early detection of emerging carbapenemase variants, track the spread of high-risk clones and assist with infection prevention. Integration of genomic data into national and international surveillance networks will strengthen monitoring of resistance dissemination and the emergence of hybrid resistance–virulence plasmids across healthcare, community, animal, and environmental settings (McArthur and Tsang, 2017).

### 6.2 Precision diagnostics and rapid resistance prediction

Current diagnostic approaches for CR-GNPs remain limited by turnaround time, cost, and incomplete characterization of resistance mechanisms. Future precision diagnostics integrating nanopore sequencing, metagenomics, CRISPR-based assays, and multiplex molecular platforms are expected to enable rapid detection of pathogens and resistance determinants directly from clinical specimens. Expanding access to affordable point-of-care (POC) molecular diagnostics, particularly in low- and middle-income countries, will improve timely diagnosis, optimize antimicrobial therapy, and strengthen antimicrobial stewardship (Elbehiry and Abalkhail, 2025).

### 6.3 AI and digital microbiology

Artificial intelligence is expected to play an increasingly important role in CR-GNP management by integrating microbiological, genomic, epidemiological, and clinical data for resistance prediction, therapeutic guidance, and outbreak surveillance. Successful implementation will require standardized datasets, robust model validation, transparent algorithms, and appropriate regulatory oversight (Peiffer-Smadja et al., 2020).

### 6.4 Novel therapeutic approaches

Novel BL/BLI combinations have expanded treatment options for CR-GNP infections; however, resistance to these agents is increasingly being reported. Continued investment in innovative therapeutics remains essential. Promising future therapeutic strategies include:

- Next-generation β-lactamase inhibitors active against MBLs
- Improved siderophore antibiotics
- Bacteriophage therapy
- Antimicrobial peptides
- Anti-virulence compounds
- Monoclonal antibody-based therapies
- Host-directed immunomodulatory approaches

Combined with rapid diagnostics and antimicrobial stewardship, these emerging therapeutic strategies have the potential to improve clinical outcomes and reduce selective pressure for AMR (Theuretzbacher et al., 2020).

### 6.5 One health and antimicrobial stewardship

Carbapenem-resistant pathogens are increasingly detected beyond healthcare settings, including environmental reservoirs, wastewater, livestock, companion animals, and the food production chain. Therefore, a One Health approach integrating human, animal, and environmental health is essential for controlling the spread of CR-GNPs. Strengthening antimicrobial stewardship across healthcare, agriculture, and veterinary medicine will be critical to reducing inappropriate antimicrobial use and slowing the emergence of AMR. National and international collaboration, harmonized surveillance, and coordinated policy implementation will be essential to address the global challenge of carbapenem resistance. Future control strategies should integrate genomic surveillance, precision diagnostics, innovative therapeutics, AI-assisted decision support, and One Health approaches to achieve sustainable control of CR-GNPs (Delpy et al., 2024; Scott et al., 2019).

## 7 Conclusion

Carbapenem-resistant Gram-negative pathogens (CR-GNPs) represent a major global health threat because of their rapid dissemination, diverse resistance mechanisms, and association with high morbidity and mortality. The widespread dissemination of carbapenemase genes through mobile genetic elements and high-risk clones has accelerated the global spread of resistance. Recent advances in rapid phenotypic assays, molecular diagnostics, whole-genome sequencing, metagenomics, and artificial intelligence have substantially improved the detection and surveillance of CR-GNPs. Novel β-lactam/β-lactamase inhibitor combinations, cefiderocol, and other targeted therapeutic approaches have expanded treatment options, although the continued emergence of resistance remains a significant challenge. Future control of CR-GNPs will require an integrated approach combining genomic surveillance, precision diagnostics, innovative therapeutics, antimicrobial stewardship, and One Health strategies to improve patient outcomes and preserve the effectiveness of current and future antimicrobial agents.
